# A retrospective study of thoracic endovascular aortic repair timing in patients with uncomplicated type B dissection who have a smoking history

**DOI:** 10.3389/fcvm.2022.1035971

**Published:** 2022-11-23

**Authors:** Hui-Qiang Gao, Guoqi Li, Hong-Kai Zhang, Lan-Lin Zhang, Shang-Dong Xu

**Affiliations:** ^1^Department of Cardiac Surgery, Beijing Anzhen Hospital, Beijing Institute of Heart Lung and Blood Vessel Diseases, Capital Medical University, Beijing, China; ^2^Beijing Institute of Heart Lung and Blood Vessel Diseases, Beijing Anzhen Hospital, Capital Medical University, Beijing, China; ^3^Department of Radiology, Beijing Anzhen Hospital, Capital Medical University, Beijing, China

**Keywords:** operative time, TEVAR, thoracic endovascular aortic repair, uncomplicated type B dissection, smoke

## Abstract

**Objective:**

To determine the optimal timing of thoracic endovascular aortic repair (TEVAR) for patients with uncomplicated type B dissections who have a smoking history.

**Methods:**

Data from 308 consecutive patients with uncomplicated type B dissections, who have a smoking history and onset-to-TEVAR time within 90 days, were analyzed. The patients were divided into two groups: Acute and subacute phases. Univariate and multivariate regression analyses were performed. Smooth curve fitting and threshold analysis were performed to characterize the relationship between the onset-to-TEVAR time and follow-up deaths.

**Results:**

There were no significant differences between the two groups. Smooth curve fitting and threshold effect analysis showed that if early TEVAR was performed within 9.4 days from onset, there was better long-term survival and there was no significant difference after 9.4 days.

**Conclusion:**

By studying the relationship between onset-to-TEVAR time and all-cause mortality, we found that early TEVAR may have a lower all-cause mortality rate during follow-up in uncomplicated type B dissection patients who have a smoking history and within 90 days from onset.

## Introduction

Thoracic endovascular aortic repair (TEVAR) is currently the preferred treatment for complicated type B dissection; however, it is still under debate whether or not TEVAR should be performed in patients with uncomplicated type B dissections and when TEVAR should be performed (1). In one of our manuscripts, we summarized the data of our center and found that the 5- and 10-year survival rates of patients with uncomplicated type B dissections who underwent TEVAR was 96.5% [95% confidence interval (CI), 95.0–98.0%] and 83.0% (95% CI, 77.9–88.4%) (2), respectively, which was significantly better than the survival rates for patients who received medical treatment, as reported in the literature (3–11). The widely accepted operative timing for patients with complicated type B dissections is as soon as possible because complicated type B dissections are often combined with factors that affect mortality, but the optimal timing for TEVAR in patients with uncomplicated type B dissections is still under debate. This study will try to use retrospective data to analyze the relationship between the time of TEVAR and long-term survival, and provide a reference for choosing TEVAR timing.

## Materials and methods

### Patients

The inclusion criteria were as follows: (1) Patients who underwent TEVAR in our hospital from May 2001 to December 2013; (2) patients with a type B dissections which has a primary tear and blood flowing in the false lumen; (3) uncomplicated type B dissections without co-morbidities, such as malperfusion, refractory hypertension, impending rupture, or rapid aortic expansion (>1 cm per year); (4) acute and subacute patients with an onset-to-TEVAR time within 90 days; and (5) patients with a smoking history before disease onset. Patients were included in this study only when five inclusion criteria were met. The exclusion criteria were as follows: (1) Patients with complicated type B dissections; (2) patients who did not undergo TEVAR due to anatomic reasons or the patient, declined TEVAR; (3) patients with aortic intramural hematoma, penetrating aortic ulcers, or other lesions who underwent TEVAR; (4) patients with connective tissue diseases, such as Marfan syndrome; (5) patients who have no smoking history; (6) patients referred to our hospital for complications of TEVAR or open procedures performed in other hospitals; or (7) patients with chronic dissection, which means the onset-to TEVAR time was > 90 days. Patients were excluded from the study when they met any one of the exclusion criteria. We defined the acute phase for patients with an onset-to-TEVAR time 14 ≤ days, the subacute phase for an onset-to-TEVAR time between 15 and 90 days, and the chronic phase for an onset-to-TEVAR time > 90 days. We defined onset-to-TEVAR time as the total time from onset-to-start time of TEVAR, which is the sum of the time from onset-to-diagnosis and the waiting time from diagnosis-to-TEVAR. We defined patients with a history of smoking as patients who had sustained smoking behavior before surgery and had a total cigarette count > 100. This study was approved by the Anzhen Hospital Ethics Committee (Beijing, China). Since this was a retrospective study, it did not require informed consent from patients.

### Thoracic endovascular aortic repair procedure

Patients completed the related examinations first, such as computed tomography angiography (CTA), and the treatment plan was determined according to the examination results. Emergency surgery was usually performed on patients with complicated type B dissections with co-morbidities, such as malperfusion or impending rupture. The timing of TEVAR for patients with uncomplicated type B dissections was considered on a case-by-case basis. TEVAR was usually performed after conservative treatment for 1–2 weeks in patients with hematomas involving the distal end of the aortic arch. For patients with type B dissections not involving the distal end of the aortic arch, the surgeon can choose conservative treatment for 1 week before TEVAR or choose an earlier TEVAR time according to his own experience. The TEVAR procedure has been reported in the previous literature (12–14). Aortic angiography was performed through a pigtail catheter to determine the locations of the lesion and the primary tear. The angiographic results were combined with the pre-operative CTA results to determine the diameter of the artery in the anchoring zone. The diameter of the stent was selected according to the oversize (5–10%). The proximal landing zone is located in zone 3 in most patients, and in a small number of patients in zone 2. In patients with the proximal landing zone in zone 2 and the stent completely occluding the left subclavian artery, the majority of patients underwent chimney TEVAR to reconstruct the left subclavian artery. If there was only one primary tear in the thoracic descending aorta, only one stent was implanted. An additional stent was placed if there were large re-entry tears in thoracic descending aorta. When the first stent was implanted, we usually do angiography again, if the distal aorta true lumen was not fully expanded, another tapered stent was implanted to fully expand the distal aorta true lumen and adapt to the taper rate of the true lumen to avoid the SINE. More often, due to the taper rate of the aortic true lumen in patients with dissection, we will implant a large tapered stent in the distal end of the first stent to accommodate the taper rate. Because the first stent often has a specific angle after placement in the true lumen, tapered stents without a longitude bar were preferred. After the stent was released, angiography was repeated to confirm that there were no endoleaks, the diameter of the distal end of the stent matched the size of the true lumen of the aorta, the distal true lumen was well-expanded, and the thoracic descending aortic segment had no major re-entry tears. The surgery was then terminated. Post-operative attention focused on whether or not there was a sensorimotor abnormality involving the lower limbs. If a risk of paraplegia existed, cerebrospinal fluid drainage was performed immediately to avoid the occurrence of paraplegia after TEVAR. Cerebrospinal fluid drainage was only performed before TEVAR when the patient was at risk for paraplegia at the same time he or she was admitted to the hospital before TEVAR.

### Follow-up

Telephone number, address, ID number, other basic information, and the name of the contact person were collected. In the first year after discharge from our hospital, patients are usually re-examined at the third and sixth months, and every year thereafter. Those patients who did not return were contacted via telephone. Patients without telephones were mailed letters. Death was considered the primary endpoint, regardless of cause.

### Statistical analysis

Continuous variables are expressed as the mean ± standard deviation or median with the associated range. Categorical variables are expressed as frequencies and percentages. Patients were divided into two groups (acute and subacute) based on the onset-to-TEVAR time. Comparisons between groups were analyzed with the chi-square test, Student’s *t*-test, or Fisher’s exact test where appropriate. Variables associated with mortality during follow-up (*P*-value < 0.1) in univariate analysis were adjusted in a multivariate regression with Cox proportional-hazards model. After adjusting for age, diabetes, and number of stents used, smooth curve fitting was performed to determine whether or not there were non-linear relationships between the onset-to-TEVAR time and follow-up deaths. For the onset-to-TEVAR time, using segmented regression with a Cox proportional-hazards model, the likelihood ratio test was used to compare the difference between models I (one-line linear regression) and II (two-piece-wise regression), and the bootstrap re-sampling method was used to analyze the threshold effect between the onset-to-TEVAR time and follow-up deaths with an adjustment for variables which change the effect value by > 10% in a covariate check. A *P*-value < 0.05 was considered statistically significant. All analyses were performed with R^1^ and Empower Stats software (X&Y Solutions, Inc., Boston, MA, USA).^2^

## Results

A total of 308 patients met the inclusion criteria and the demographic information for the 308 patients are listed in Table 1. Three patients developed paraplegias during the peri-operative period. Two patients died peri-operatively and they were all died of aortic rupture caused by retrograde type A dissections. There were no cerebral infarctions post-operatively. There was no significant difference in the incidence of adverse events between groups.

**TABLE 1:** Demographic data of the 308 patients.

Variable	Acute phase	Subacute phase	*P*-value
*N*	207	101	
Age	52.0 ± 10.8	52.4 ± 10.4	0.781
Gender (male)	201 (97.1%)	97 (96.0%)	0.622
BMI	26.8 ± 4.2	25.7 ± 3.7	0.055
Hypertension	150 (72.5%)	78 (77.2%)	0.371
CAD	8 (3.9%)	7 (6.9%)	0.241
Diabetes	5 (2.4%)	6 (5.9%)	0.118
Drinking	81 (39.1%)	40 (39.6%)	0.936
**Number of stents used**			0.537
1	182 (88.8%)	86 (85.1%)	
2	20 (9.8%)	14 (13.9%)	
3	3 (1.5%)	1 (1.0%)	
**Stent brand**			0.061
Talent	2 (1.0%)	7 (6.9%)	
Valiant	53 (25.6%)	29 (28.7%)	
GRIMED	58 (28.0%)	19 (18.8%)	
Hercules	30 (14.5%)	17 (16.8%)	
Zenith TX2	28 (13.5%)	10 (9.9%)	
Relay	23 (11.1%)	11 (10.9%)	
E-vita	13 (6.3%)	8 (7.9%)	
Hybrid	1 (0.5%)	2 (2.0%)	0.209
Chimney TEVAR	9 (4.3%)	6 (5.9%)	0.542

Values are presented as the mean ± standard deviation or *n* (%).

BMI, body mass index; CAD, coronary artery disease; TEVAR, thoracic endovascular aortic repair.

**FIGURE 1:**
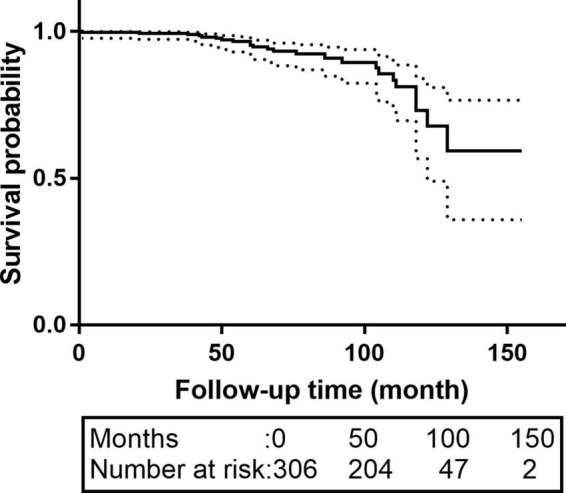
The K-M survival curves of patients with acute and subacute uncomplicated type B dissection who have a history of smoking. The 5-and 10-year survival rates were 95.1% (95% CI: 92.3–98.1%) and 73.1% (95% CI: 60.7–87.9%), respectively. CI, confidence interval.

**FIGURE 2:**
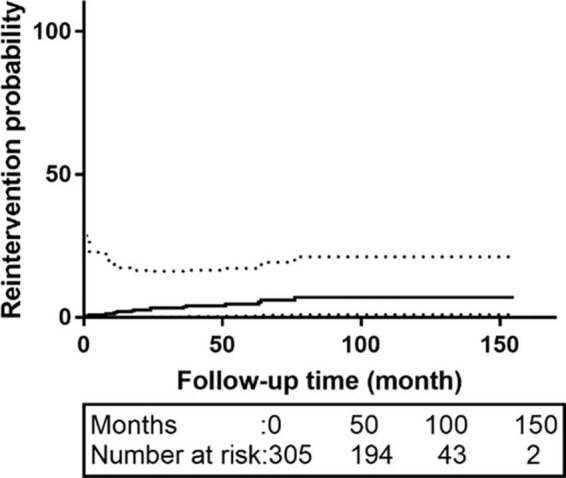
The re-intervention curves of patients with acute and subacute uncomplicated type B dissection who have a history of smoking. The 5-and 10-year re-intervention rates were 5.3% (95% CI: 2.6–7.9%) and 7.6% (95% CI: 3.8–11.2%), respectively. CI, confidence interval.

Sixty-three patients were lost to follow-up; the rate of loss was 20.6%. The median follow-up time was 62.0 months [interquartile range (IQR):42.0–83.0 months]. The K-M survival curve of patients during follow-up are shown in Figure 1. The 5- and 10-year survival rates were 95.1% (95% CI: 92.3–98.1%) and 73.1% (95% CI: 60.7–87.9%), respectively. During follow-up, 16 patients received intervention again (retrograde type A dissection, 4; stent-induced new entry [SINE], 5; endoleaks, 2; aneurysm formation, 2; and residual dissection, 3), 5 of whom underwent open surgery and 11 of whom were treated with TEVAR again. The patient re-intervention curve is shown in Figure 2. The 5- and 10-year re-intervention rates were 5.3% (95% CI: 2.6–7.9%) and 7.6% (95% CI: 3.8–11.2%), respectively.

**FIGURE 3:**
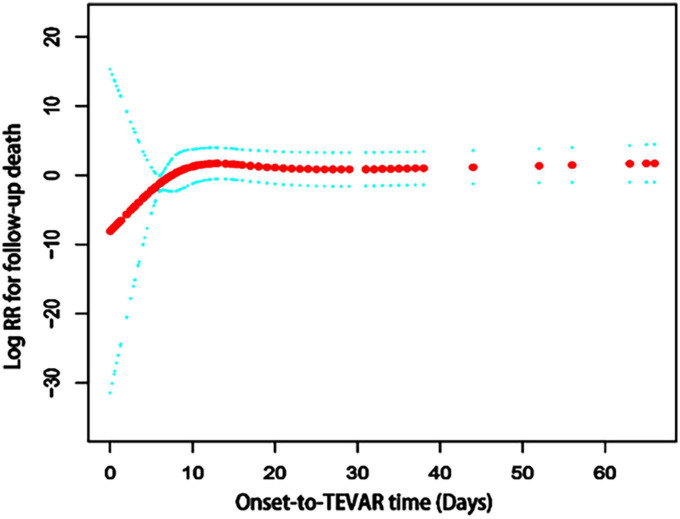
The spline smoothing plot between onset-to-TEVAR time and follow-up death risk in acute and subacute uncomplicated type B dissection patients who have a history of smoking. The results showed that the curve was elevated in the early stage of the disease, thus the long-term death risk increased with a delay in the onset-to-TEVAR time. When the onset-to-TEVAR time was delayed beyond a specific inflection point (9.4 days), the curve became a near-horizontal state, which indicates that the long-term death risk no longer increased with the delay in onset-to-TEVAR time. RR, Relative risk; TEVAR, thoracic endovascular aortic repair.

The results of univariate analysis are shown in Table 2. The results of the multivariable analyses use Cox proportional hazards model are shown in Table 3. After adjustment for age, diabetes, number of stents used, the hazard ratio (HR) of the acute group (onset-to-TEVAR time <14 days) was 0.7 (95% CI: 0.3–1.5; *P* = 0.348) compared with the subacute phase group (onset-to-TEVAR time within 15–90 days). Compared with the subacute phase, although the *P*-value was not significant, it appears that TEVAR in the acute phase was more conducive to long-term survival. Therefore, smooth curve fitting was performed to determine whether or not there were non-linear relationships between the onset-to-TEVAR time and follow-up deaths. The curve fitting results are shown in Figure 3. The results show the risk of death during follow-up as a function of time increased with the delay of the operative time within a specific turning point. When the curve exceeded the turning point, the fitted figure was close to a straight line. Therefore, we performed a threshold effect analysis. After adjusting for age, BMI, hypertension, coronary artery disease (CAD), stent brand, chimney TEVAR, and variables that changed the effect values by > 10%, we found that the risk of death during the follow-up period increased following a delay of TEVAR within 9.4 days from the onset time (HR:7.8; 95% CI:0.5–120.5), and the risk remained unchanged after 9.4 days (HR: 1.0; 95% CI: 1.0–1.0; *P* = 0.002), as shown in Table 4.

**TABLE 2:** Univariate analysis results.

Variable	Statistics	HR (95%CI) *P*-value
TEVAR timing	21.2 ± 24.4	1.0 (1.0, 1.0) 0.097
Age	52.0 ± 10.6	1.1 (1.0, 1.1) 0.002
Gender (female)	10 (3.3%)	2.5 (0.6, 10.9) 0.210
BMI	26.4 ± 4.1	1.0 (0.8, 1.1) 0.481
Hypertension	226 (73.9%)	1.0 (0.4, 2.4) 0.996
CAD	15 (4.9%)	0.7 (0.1, 5.3) 0.745
Diabetes	11 (3.6%)	3.2 (1.0, 10.9) 0.058
Alcohol consumption	121 (39.5%)	1.3 (0.6, 3.0) 0.462
**Number of stents used**		
1	266 (87.5%)	1
2	34 (11.2%)	2.6 (1.0, 6.9) 0.060
3	4 (1.3%)	0.0 (0.0, Inf) 0.998
**Stent brand**		
Talent	9 (2.9%)	1
Valiant	82 (26.8%)	0.7 (0.1, 4.5) 0.734
GRIMED	75 (24.5%)	1.2 (0.2, 5.5) 0.856
Hercules	47 (15.4%)	1.6 (0.3, 7.7) 0.575
Zenith TX2	38 (12.4%)	1.0 (0.1, 12.2) 0.998
Relay	34 (11.1%)	1.6 (0.2, 10.7) 0.627
E-vita	21 (6.9%)	1.4 (0.1, 16.4) 0.812
Hybrid	3 (1.0%)	0.0 (0.0, Inf) 0.998
Chimney TEVAR	15 (4.9%)	0.0 (0.0, Inf) 0.997

Values are presented as the mean ± standard deviation or n (%) or HR (95% CI) *P*-value. BMI, body mass index; CAD, coronary artery disease; TEVAR, thoracic endovascular aortic repair.

**TABLE 3:** Multivariate regression results of TEVAR in different stages.

Staging	Non-adjusted	Adjust
Subacute phase	1	1
Acute phase	0.6 (0.3, 1.4) 0.220	0.7 (0.3, 1.5) 0.348

Multivariate regression used the Cox proportional-hazards model. Values are presented as the HR (95% CI) *P*-value. Non-adjusted model adjusted for: None. Adjust model adjusted for: age, diabetes, and number of stents used.

**TABLE 4:** Threshold analysis for the relationship between onset-to-TEVAR time and follow-up death risk.

Models	Adjusted HR (95% CI) *P*-value
**Model I**	
One line slope	1.0 (1.0, 1.0) 0.288
**Model II**	
Turning point (K)	9.4
<K slope 1	7.8 (0.5, 120.5) 0.142
>K slope 2	1.0 (1.0, 1.0) 0.685
LRT test	0.002*

Values are presented as the HR (95% CI) P-value. Adjusted for: age, BMI, hypertension, CAD, diabetes, stent brand, chimney TEVAR; LRT test: logarithmic likelihood ratio test.

*Indicates that Model II is significantly different from Model I.

## Discussion

Emergency surgery is not controversial for patients with complicated type B dissections because complicated type B dissections often have life-threatening complications; however, there is no consensus with respect to when TEVAR should be performed in patients with uncomplicated type B dissections (1). Surgeons often choose the appropriate timing of TEVAR based on their own experience, but there is a lack of direct evidence to prove the best time. Our data may fill in this gap of knowledge. According to our analysis, the earlier TEVAR is performed in patients within 9.4 days of onset, the better the long-term mortality risk; however, there was no significant difference after 9.4 days of onset.

Unlike international physicians who only perform TEVAR for patients with high-risk factors or in patients whose aortic diameter expands rapidly, we also actively perform TEVAR for patients with subacute and chronic dissections. Because patients in China have a longer life expectancy and poor blood pressure control, we tend to intervene as early as possible. For patients with subacute and chronic type B dissections, when the diameter of the descending aorta is < 5 cm, especially when the diameter of the true lumen is < 4.5 cm, TEVAR can still be performed. At this time, the diameter of the stent should be considered according to the diameter of the anchoring zone and the true lumen. The main purpose of the treatment in chronic patients is to close the primary tear and prevent the blood from continuously flowing into the false lumen, which may lead to further expansion or even rupture of the false lumen. When patients develop a thoracic and abdominal aortic aneurysm, most patients will undergo thoracic and abdominal aortic replacement; however, when the patient’s general condition is not suitable for surgery and the diameter of the true lumen is small, TEVAR can still be considered to reduce the possibility of aneurysm rupture. Patients with acute type B dissections rarely form aneurysms. If the aortic diameter is large, there is generally only a large single primary tear without a re-entry tear, therefore the false lumen pressure cannot be released, so there is rapid expansion. At this time, TEVAR treatment is still preferred. When the primary tear is sealed, the aortic diameter can be slowly reduced after the false lumen pressure is reduced.

The debate about whether or not patients with uncomplicated type B dissections should undergo TEVAR continues (15–17), but there is increasing evidence supporting the opinion that TEVAR may be more beneficial for the long-term prognosis of patients with uncomplicated type B dissections (18–20). There is also no consensus about the optimal timing for TEVAR among patients with uncomplicated type B dissections (21–23). There are two important questions with respect to optimal timing for TEVAR. First, when should TEVAR be performed to minimize the peri-operative mortality and complication rate? Second, what timing is more conducive to long-term survival? In our center, TEVAR is usually performed at 7–14 days, especially for patients with hematomas involving the distal end of the aortic arch, because we believe that for these patients retrograde type A dissection or stent-induced new entry (SINE) is more likely to occur. After medical treatment for 1–2 weeks, the risk of retrograde type A dissection is relatively lower after absorption of the hematoma. Therefore, it is best to perform surgery between 7 and 14 days for such patients; however, for a uncomplicated type B dissection without hematoma involving the distal end of the aortic arch, whether or not the time for TEVAR is selected between 7 and 14 days should consider the relationship between TEVAR timing and the long-term survival rate. Our center has performed TEVAR on patients with uncomplicated type B dissections since 2001, thus there is a large amount of data on the timing and long-term survival. According to our analysis, patients undergoing TEVAR during the early stage will have a lower risk of long-term mortality than patients has a long onset-to-TEVAR time, especially in patients within 9.4 days of onset. This result implied that TEVAR should be performed as early as possible for uncomplicated type B dissections, but actually we cannot perform TEVAR early for all patients. The surgeon should first assess the risk of serious adverse events during the peri-operative period. When the CTA shows a hematoma involving the distal end of the aortic arch leading to the possibility of retrograde type A dissection, it is necessary to conservative treatment for 7–14 days before TEVAR. For patients at low risk, TEVAR can be performed as soon as possible.

The reason why early TEVAR can reduce the risk of long-term mortality may be that early TEVAR can better promote aortic remodeling (24). The intima of the aorta is more likely to become stiff in smoking patients, so the earlier the TEVAR, the better the intimal compliance, the better the remodeling of the aorta, and the better the long-term prognosis. However, this conjecture still needs further verification because some studies have also proposed the opposite view that early surgery is a risk factor for aortic dilation (21). Unfortunately, our study lacked support from follow-up imaging data, so it was only possible to analyze all-cause mortality during the follow-up period and we could not further analyze the impact of TEVAR timing on aortic remodeling. This study was a single center retrospective study and therefore had some limitations. Although the TEVAR time of our patients was distributed over various time periods, this was already an artificially selected distribution. For example, patients with hematomas involving the distal end of the aortic arch usually underwent surgery 7–14 days of onset. Fortunately, we believe that this selectivity has limited impact on the conclusions of this study. Because this selective delay in onset-to-TEVAR time reduces the feasibility of perioperative complications, the relationship between delayed onset-to-TEVAR time and long-term mortality rates is still a one-to-one relationship. Therefore, we believe that this delay had no significant impact on this correspondence and our conclusion. Due to China’s medical system, the post-operative image re-examination rate of Chinese patients is not satisfactory, which led to some patients who need to undergo re-intervention did not actually undergo re-intervention because the patients did not know that their disease progressed. Therefore, the re-intervention curve given in this study may be lower than the proportion of patients who actually need re-intervention. Another regret of this paper is that the DISSECT and TEM classification was not used to classify the patients. Data entry for this paper started in 2015 and ended in 2016. There was no TEM classification (25) when this database was designed, and the standards of DISSECT (26) were not designed into this database when we designed it, so this part of the classification data is missing, and the patients in this article cannot be classified by these two methods. Because the data in this article is relatively old, there are some missing data, and the description in this article cannot fully meet the reporting standards required in the latest literature (27–29). The lost-to-follow-up rate in this study was high, reaching 20.6%, which may have some impact on the accuracy of the results, and it is unclear whether this impact is beneficial to the conclusion. The study population was Chinese. The patients in China with dissections were younger and the blood pressure control was not optimal. Therefore, if the patients with uncomplicated type B dissections were treated conservatively, the aorta would more likely be dilated. Then, patients with uncomplicated type B dissections in China would routinely undergo TEVAR without waiting for co-morbidities or aortic dilation. Our data showed that the sooner TEVAR was performed, the higher the long-term survival rate; however, due to the characteristics of the Chinese patients, whether or not this conclusion is applicable to European and American patients remains to be verified. Further research is needed to confirm the optimal TEVAR timing for patients with uncomplicated type B dissections.

## Conclusion

Within 9.4 days of onset, the earlier TEVAR is performed, the better it is for long-term survival for patients with uncomplicated type B dissections, but the risk remained unchanged after > 9.4 days. Early surgery is not recommended for all patients. When there are risk factors, such as a hematoma involving the distal end of the aortic arch leading to high risk for retrograde type A dissection or SINE, conservative treatment for 7–14 days is recommended before TEVAR.

## Data availability statement

The raw data supporting the conclusions of this article will be made available by the authors, without undue reservation.

## Ethics statement

The studies involving human participants were reviewed and approved by the Anzhen Hospital Ethics Committee. Written informed consent for participation was not required for this study in accordance with the national legislation and the institutional requirements.

## Author contributions

H-QG, GQL, H-KZ, L-LZ, and S-DX: conception and design, analysis and interpretation, drafting of the manuscript, and revising the manuscript for intellectual content. All authors have read and approved the final manuscript.

## Footnotes

1
https://www.r-project.org/

2
https://www.empowerstats.net/en/
